# Associations of Regional Brain Structural Differences With Aging, Modifiable Risk Factors for Dementia, and Cognitive Performance

**DOI:** 10.1001/jamanetworkopen.2019.17257

**Published:** 2019-12-11

**Authors:** Hideaki Suzuki, Ashwin V. Venkataraman, Wenjia Bai, Florian Guitton, Yike Guo, Abbas Dehghan, Paul M. Matthews

**Affiliations:** 1 Tohoku Medical Megabank Organization, Tohoku University, Sendai, Japan; 2 Department of Cardiovascular Medicine, Tohoku University Graduate School of Medicine, Sendai, Japan; 3 Division of Brain Sciences, Department of Medicine, Imperial College London, London, United Kingdom; 4 United Kingdom Dementia Research Institute, London, United Kingdom; 5 Data Science Institute, Imperial College London, London, United Kingdom; 6 Department of Epidemiology and Biostatistics, Imperial College London, London, United Kingdom

## Abstract

**Importance:**

Identifying brain regions associated with risk factors for dementia could guide mechanistic understanding of risk factors associated with Alzheimer disease (AD).

**Objectives:**

To characterize volume changes in brain regions associated with aging and modifiable risk factors for dementia (MRFD) and to test whether volume differences in these regions are associated with cognitive performance.

**Design, Setting, and Participants:**

This cross-sectional study used data from UK Biobank participants who underwent T1-weighted structural brain imaging from August 5, 2014, to October 14, 2016. A voxelwise linear model was applied to test for regional gray matter volume differences associated with aging and MRFD (ie, hypertension, diabetes, obesity, and frequent alcohol use). The potential clinical relevance of these associations was explored by comparing their neuroanatomical distributions with the regional brain atrophy found with AD. Mediation models for risk factors, brain volume differences, and cognitive measures were tested. The primary hypothesis was that common, overlapping regions would be found. Primary analysis was conducted on April 1, 2018.

**Main Outcomes and Measures:**

Gray matter regions that showed relative atrophy associated with AD, aging, and greater numbers of MRFD.

**Results:**

Among 8312 participants (mean [SD] age, 62.4 [7.4] years; 3959 [47.1%] men), aging and 4 major MRFD (ie, hypertension, diabetes, obesity, and frequent alcohol use) had independent negative associations with specific gray matter volumes. These regions overlapped neuroanatomically with those showing lower volumes in participants with AD, including the posterior cingulate cortex, the thalamus, the hippocampus, and the orbitofrontal cortex. Associations between these MRFD and spatial memory were mediated by differences in posterior cingulate cortex volume (β = 0.0014; SE = 0.0006; *P* = .02).

**Conclusions and Relevance:**

This cross-sectional study identified differences in localized brain gray matter volume associated with aging and MRFD, suggesting regional vulnerabilities. These differences appeared relevant to cognitive performance even among people considered cognitively healthy.

## Key Points

**Question:**

Are aging and modifiable risk factors for dementia (MRFD) associated with volume differences in brain regions also associated with Alzheimer disease (AD)?

**Findings:**

In this cross-sectional study including 8312 participants, 4 MRFD (hypertension, diabetes, obesity, and frequent alcohol use) were independently and negatively associated with overlapping gray matter regions, including the posterior cingulate cortex, which overlapped with regions that are known to show atrophy in AD. The negative associations of MRFD with spatial memory were associated with mediation through differences in posterior cingulate cortex volume.

**Meaning:**

These findings suggest that common brain regions associated with change with aging and MRFD may explain their joint contributions to cognitive variances in people considered cognitively healthy and in people with AD.

## Teaser

This cross-sectional study examines the associations of aging and modifiable risk factors for dementia with volume differences in brain regions known to be associated with Alzheimer disease and whether these volume differences are associated with cognitive performance.

## Introduction

The prevalence of dementia, estimated in most countries to be 5% to 7% among people 60 years and older, is increasing globally with greater longevity.^1^ A likely contributor to rising incidence in dementia other than aging is the global increase in lifestyle diseases, such as hypertension, diabetes, and obesity.^2,3,4^ These and other modifiable risk factors for dementia (MRFD), such as alcohol use,^5^ additively contribute approximately one-third of the total risk of Alzheimer disease (AD),^6,7^ although the pathophysiological mechanisms are not well understood.

Brain atrophy is the most prominent characteristic of brain structural abnormalities in AD. Subtle structural differences similar to those later expressed with AD can be identified in the brains of people who are cognitively healthy (CH) at least a decade before any expected onset of AD.^8^ Atrophy in brain regions independently associated with early pathology of AD is associated with cognitive decline in individuals who are otherwise CH.^9^ These and related observations suggest that brain structure is sensitive to factors contributing to late-life cognitive impairment and dementia.

The brain also atrophies with increasing age^10,11,12,13,14^ at an mean rate of 0.5% per year after age 40 years, although rates of volume loss vary considerably between individuals.^10,15,16^ Possible explanations for these differences include both genetic variation and differences in exposures to environmental or lifestyle factors that themselves accelerate brain atrophy. Numerous neuroimaging experiments have revealed that the brain atrophy is accelerated by hypertension,^13,17,18,19^ diabetes,^20,21,22,23,24^ obesity,^25,26^ smoking,^27,28^ low educational attainment,^29,30^ high alcohol intake,^31,32^ and inadequate sleep.^33,34^ However, whether aging and MRFD only influence brain regions sensitive to earlier neuropathological changes with AD or broadly affect all regions of the brain still remains unclear.

We addressed this question using data from the UK Biobank^35,36^ (UKB) and Alzheimer’s Disease Neuroimaging Initiative (ADNI). The UKB^35,36^ is a resource for prospective, longitudinal brain magnetic resonance images (MRIs) associated with detailed health and lifestyle information characterizing large numbers of people recruited from general practice registers across the United Kingdom.^36^ The primary purpose of our study was to determine whether aging and MRFD had independent associations with regional gray matter volumes (GMVs) also associated with AD. We also tested whether associations of aging and multiple MRFD on cognitive impairment are associated with mediation by these regional GMV differences.

## Methods

### Study Participants

The UKB recruited approximately 500 000 participants aged 40 to 69 years across Great Britain between March 13, 2006, to October 1, 2010.^35^ Since 2014, a subset of participants underwent brain MRI.^36^ For this cross-sectional study, we used a data set that included participants who underwent structural brain MRI from August 5, 2014, to October 14, 2016. The UKB received National Research Ethics Approval. All participants provided written informed consent. This study was conducted under terms of UKB access approval and is reported following the Strengthening the Reporting of Observational Studies in Epidemiology (STROBE) reporting guideline.

To characterize gray matter regions associated with AD, we used brain MRIs from 100 patients with AD and 133 age-, sex-, and race/ethnicity-matched healthy controls contributing to the ADNI resource.^37^ The ADNI was launched in 2003 as a public-private partnership, led by Michael W. Weiner, MD. The primary goal of ADNI has been to determine whether serial MRI, positron emission tomography, other biological markers, and clinical and neuropsychological assessments can be combined to measure the progression of mild cognitive impairment and early AD. The ADNI received US ethical approval from 58 study locations, with all participants providing written informed consent. Inclusion and exclusion criteria for the ADNI cohort have been described previously.^37^

### Baseline Characteristics

Information on age, sex, race/ethnicity, MRFD (ie, hypertension, diabetes, obesity, frequency of alcohol use, current smoking, and sleep duration), educational attainment, and history of neurological or psychiatric disease were reported by UKB participants at the time of the imaging assessment. Definitions of MRFD are provided in the eAppendix in the Supplement.^25,26,38,39,40,41^ Information on histories of neurological or psychiatric diseases was obtained from the *International Statistical Classification of Diseases and Related Health Problems, Tenth Revision* ^42^ codes in the UKB database (*F0-509*, *F540*, *F590*, *F700-890*, *F990*, *G40-119*, *G130-239*, *G300-419*, *G460-468*, *G800-948*, *G972*, *I600-I698*, and *I780*). Data on age, sex, and race/ethnicity also were collected from the ADNI database.^37^

### Cognitive Assessment

We used the number of uncorrected matches in the pair matching test,^43^ the mean time to correctly identify matches in the reaction time test,^44^ and the number of questions attempted within the time limit for assessment of spatial memory, reaction time, and fluid intelligence^45^ as described previously^46^ (see eAppendix in the Supplement). Cognitive assessment data were acquired on the same day of brain MRI acquisition.

### Brain MRI Acquisition and Analysis

The MRI acquisition and processing are described in the eAppendix in the Supplement).^47,48,49,50,51,52^ Briefly, gray matter probabilistic maps were segmented from acquired UKB and ADNI T1 MRIs and normalized to the same standard Montreal Neurological Institute space for voxelwise analyses.

### Statistical Analysis

All analyses were performed using Stata statistical software version 14 (StataCorp) unless otherwise stated. Distributions of continuous variables are shown in eFigure 1 in the Supplement. Normally distributed continuous variables were analyzed using the *t* test, continuous variables which were not distributed normally were analyzed using the Mann-Whitney test, and nominal variables were analyzed using the Pearson χ^2^ statistic. *P* values were 2-tailed, and significance levels were set at less than .05 unless otherwise indicated. Familywise error rate (FWER)–corrected *P* values less than .05 were used as a significance threshold in voxelwise analyses owing to multiple voxel comparisons.^53^

We first demonstrated independent associations of age and MRFD with total GMV in the UKB cohort (eAppendix in the Supplement). The correlation coefficients across age, MRFD, and the other covariates are shown in eTable 1 in the Supplement. To define the gray matter regions in which volume differences were associated with aging and the MRFD, we performed a voxelwise linear mixed analysis^51,54^ using a model in which each voxel of the normalized gray matter maps was a dependent variable and age and MRFD were independent variables adjusted for sex, race/ethnicity, educational attainment, and intracranial volume (ICV). We separately contrasted GMVs in the ADNI AD and CH groups on a voxelwise basis to create a binarized mask describing regions showing relative atrophy with AD. These 2 voxelwise analyses in a common brain space allowed for testing whether gray matter regions in which volume differed with aging and MRFD in the UKB population overlapped with those associated with regional atrophy in AD for the ADNI population. This primary analysis was conducted on April 1, 2018.

We also tested whether the association between age and GMV was modified by MRFD in the UKB cohort by entering the product of age and the number of MRFD in the linear regression model with total GMV as the outcome. This interaction was visualized using regressions between normalized total GMV and age for variable numbers of MRFD. Normalized total GMV was calculated for each regression relative to the total GMV to ICV ratio at age 50 years.

Finally, we tested whether associations of aging and multiple MRFD with cognitive impairment were associated with mediation by their brain volume differences using structural equation modeling. Before conducting structural equation modeling, we tested associations of cognitive test scores with brain regions showing differences with aging and MRFD (eAppendix in the Supplement). Associations of aging and MRFD with cognitive test scores also were tested (eAppendix in the Supplement). After testing these associations, structural equation modeling was used to test for mediation of the association of age and numbers of MRFD with spatial memory score by the volume of the posterior cingulate cortex. We chose to explore this mediation using spatial memory score and posterior cingulate cortex volume because of the observed associations of spatial memory with both the number of MRFD and posterior cingulate cortex volume. Direct associations of the MRFD with cognitive function also were tested. Goodness of the model fit was tested using χ^2^, root mean square error of approximation, Akaike information criterion, comparative fit index, and Tucker-Lewis index.

## Results

### Characteristics of Participants Selected

Among 9932 participants recruited from the UKB aged 40 to 69 years who had brain MRI data, we excluded 507 individuals lacking cognitive test data, 628 lacking baseline characterization, and 485 individuals with neurological or psychiatric disease. Characteristics of the remaining 8312 participants (mean [SD] age, 62.4 [7.4] years; 3959 [47.1%] men) whose brain images were used in this study are described in the Table. Compared with included participants, the UKB imaging cohort participants whose data were available but who were excluded were older by a mean of 1.2 years (95% CI, 0.8-1.6 years; *P* < .001), were less likely to be white (8097 white participants [97.4%] vs 1518 white participants [95.8%]; *P* = .001), and had higher prevalences of risk factors for late-life cognitive impairment, including hypertension (4236 participants [49.0%] vs 718 participants [63.1%]; *P* < .001), diabetes (400 participants [4.8%] vs 114 participants [7.5%]; *P* <.001), obesity (1515 participants [18.2%] vs 385 participants [24.2%]; *P* < .001), current smoking (286 participants [3.4%] vs 126 participants [16.6%]; *P* < .001), and inadequate sleep (986 participants [11.6%] vs 272 participants [17.8%]; *P* < .001), and had lower prevalence of college education (3936 participants [47.4%] vs 564 participants [37.2%]; *P* < .001). Additionally, compared with the included participants, excluded participants had a lower median (interquartile range [IQR]) score for spatial memory (3 [0-5] vs 2 [0-4]; *P* < .001), higher median (IQR) reaction time (566 [511-636] milliseconds vs 577 [520-655] milliseconds; *P* < .001), a lower mean (SD) fluid intelligence score (8.82 [2.09] vs 8.64 [2.14]; *P* < .001), and lower mean ICV volume (difference, 11.5 mL; 95% CI, 3.5-19.5 mL; *P* = .005), total brain volume, (difference, 20.4 mL; 95% CI, 14.5-26.2 mL; *P* < .001), total GMV (difference, 13.5 mL; 95% CI, 9.9-17.0 mL; *P* < .001), and total white matter volume (difference, 6.9 mL; 95% CI, 4.1-9.7 mL; *P* < .001).

**Table.** Participants Characteristics

Characteristic	UK Biobank Cohort, No. (%)		ADNI Cohort, No. (%)	
	Included Participants, No. (%) (n = 8312)	Excluded Participants, No./Total No. (%) (n = 1620)	Patients With AD (n = 100)	Individuals Who Were CH (n = 133)
Nonmodifiable factors				
Age, mean (SD), y	62.4 (7.4)	63.6 (7.7)	74.6 (8.1)	73.4 (6.3)
Men	3959 (47.6)	770/1620 (47.5)	57 (57.0)	65 (48.9)
White	8097 (97.4)	1518/1584 (95.8)	117 (89.0)	89 (88.0)
Intracranial volume, mean (SD), mL	1547.5 (149.7)	1536.0 (151.6)	1500.1 (169.5)	1488.3 (148.6)
Overall brain volume measures, mean (SD), mL				
Total brain	1120.4 (110.0)	1100.0 (113.5)	955.5 (112.6)	1013.9 (116.8)
Total gray matter	664.6 (67.0)	651.2 (68.9)	557.4 (71.9)	604.4 (75.3)
Total white matter	455.7 (52.9)	448.8 (54.5)	398.1 (51.0)	409.4 (52.1)
Modifiable risk factors for dementia				
Hypertension	4236 (49.0)	718/1138 (63.1)	NA	NA
Diabetes	400 (4.8)	114/1530 (7.5)	NA	NA
Obesity	1515 (18.2)	385/1590 (24.2)	NA	NA
Current smoking	286 (3.4)	126/1527 (8.3)	NA	NA
Frequent alcohol use	1478 (17.9)	257/1549 (16.6)	NA	NA
Inadequate sleep	968 (11.6)	272/1532 (17.8)	NA	NA
Cognitive test scores and educational attainment				
Mini-Mental State Examination score, median (IQR)	NA	NA	29 (29-30)	23 (21-25)
Spatial memory score, median (IQR)	3 (0-5)	2 (0-4)^a^	NA	NA
Reaction time, median (IQR), ms	566 (511-636)	577 (520-655)^b^	NA	NA
Fluid intelligence score, mean (SD)	8.82 (2.09)	8.64 (2.14)^c^	NA	NA
College degree education	3936 (47.4)	564 (37.2)^d^		
Cerebral amyloid standardized uptake value, median (IQR)	NA	NA	1.05 (1.00-1.18)^e^	1.43 (1.25-1.54)^f^

Abbreviations: AD, Alzheimer disease; ADNI, Alzheimer’s Disease Neuroimaging Initiative; CH, cognitively healthy; IQR, interquartile range; NA, not available.

^a^ Includes data from 1550 participants.

^b^ Includes data from 1501 participants.

^c^ Includes data from 1135 participants.

^d^ Includes data from 1518 participants.

^e^ Includes data from 97 participants.

^f^ Includes data from 128 participants.

The Table also presents characteristics of patients with AD and age-, sex-, and race/ethnicity-matched individuals who were CH selected from the ADNI cohort to develop a gray matter image mask describing regions showing relative atrophy with AD. Lower total brain volume in individuals with AD compared with individuals who were CH (difference, 60.0 mL; 95% CI, 31.4-88.7 mL; *P* < .001) was explained by lower total GMV alone (difference, 48.5 mL; 95% CI, 30.3-66.7 mL; *P* < .001); the difference in total white matter volume between the groups was not significant (difference, 11.6 mL; 95% CI, –3.0 to 26.1 mL; *P* = .12). Consistent with their diagnoses, participants with AD, compared with participants who were CH, had a lower a mean Mini-Mental State Examination score (difference, 6.0; 95% CI, 5.0-7.0; *P* < .001) and higher mean cerebral amyloid standardized uptake value (difference, 0.31; 95% CI, 0.25-0.37; *P* < .001).

### Overlap of Regions Associated With AD With GMV Differences Explained by Aging and MRFD 

**Figure 1.**
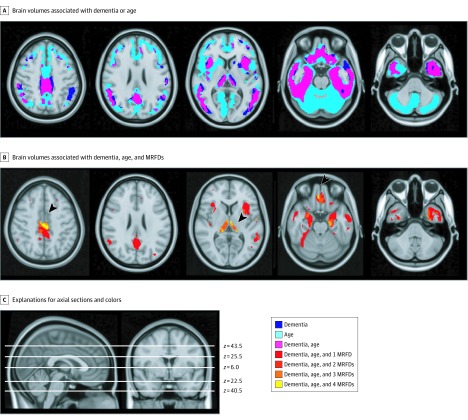
Gray Matter Regions With Volumes Associated With Alzheimer Disease, Age, and Modifiable Risk Factors for Dementia (MRFD) A, Purple regions define the overlap between gray matter regions showing lower volume associated with Alzheimer disease in the Alzheimer’s Disease Neuroimaging Initiative cohort (dark blue) and those associated with lower volumes with age in UK Biobank (light blue). B, Gray matter regions showing lower volume with dementia, greater age, and 1 (red), 2 (bright red), 3 (orange) or 4 (yellow) MRFD; the arrowheads indicate regions in the posterior cingulate cortex, the thalamus, the hippocampus, and the orbitofrontal cortex influenced by all of these factors (yellow). C, Positions of the axial slices selected for representations in A and B are shown.

We generated an image mask describing the distributions of regional brain volume differences in people with AD compared with people who were CH in the ADNI cohort. A voxelwise analysis showed that the differences were predominantly in the gray matter of the temporal, prefrontal, and posterior cingulate cortices (FWER-corrected *P* < .05) (Figure 1A). Based on this observation, analyses of the associations of aging and MRFD with brain structure focused on analyses of GMV differences in the UKB cohort.

Before the regional analysis, we tested for the independent associations of aging and 6 MRFD with total GMV of participants in the UKB cohort. Greater age (b = –3.54 mL; SE = 0.06 mL; *P* < .001) and all MRFD, including hypertension (b = –5.01 mL; SE = 0.96 mL; *P* < .001), diabetes (b = –14.39 mL; SE = 2.12 mL; *P* < .001), obesity (b = –4.70 mL; SE = 1.19 mL; *P* < .001), frequent alcohol use (b = –4.90 mL; SE = 1.18 mL; *P* < .001), current smoking (b = –6.06 mL; SE = 2.46 mL; *P* = .01), and inadequate sleep (b = –2.91 mL; SE = 1.39 mL; *P* = .04), were associated with lower GMV.

**Figure 2.**
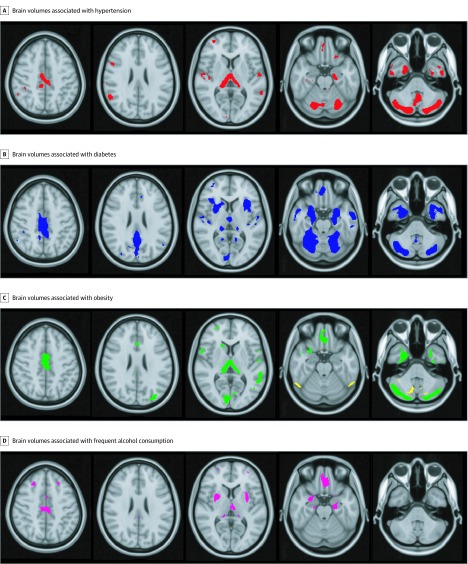
Gray Matter Regions With Volumes Associated With Individual Modifiable Risk Factors for Dementia Gray matter regions showing lower volume with hypertension (red) (A), lower volume with dementia (blue) (B), lower (green) and higher (yellow) volume with obesity (C), and lower volume with frequent alcohol use (magenta) (D).

We then conducted a voxelwise regression analysis to spatially map these independent associations of age and those of each MRFD. Age-associated differences were found across most of the gray matter (FWER-corrected *P* < .05) (Figure 1A). These differences extended beyond the gray matter regions in which volume differences associated with AD were found in the ADNI cohort, that is, only 84% of gray matter showing differences associated with age also showed differences associated with AD. Sex-associated differences in regional brain volume are shown in eFigure 2 in the Supplement (FWER-corrected *P* < .05). Gray matter regions in which volume was lower in men compared with women overlapped those associated with AD and with age. Individual brain regions in which volume was associated with hypertension, diabetes, obesity, or frequent alcohol use are presented in Figure 2. Lower GMV associated with these 4 MRFD (ie, hypertension, diabetes, obesity, and frequent alcohol use) (Figure 2) mapped within the larger volume showing GMV differences associated with age (FWER-corrected *P* < .05) (Figure 1B) and overlapped in the posterior cingulate cortex, the thalamus, the hippocampus, and the orbitofrontal cortex (Figure 1B). Neuroanatomically different gray matter regions showed negative volume associations with smoking or self-reported inadequate sleep. While the overall association of obesity with GMV was negative, smaller brain regions were found in which obesity was associated with higher GMV.

### Interaction of Aging With MRFD

**Figure 3.**
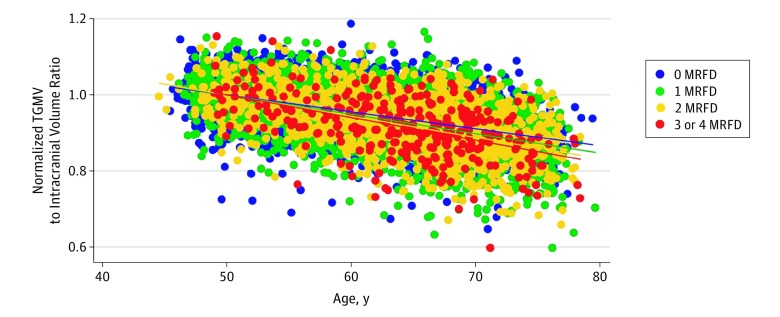
Normalized Regressions of the Variation in Overall Gray Matter Volume Associated With Age by Number of Modifiable Risk Factors for Dementia (MRFD) Normalized total gray matter volume (TGMV) was calculated for each regression relative to the total gray matter to intracranial volumes ratio at age 50 years. Dots indicate individual normalized values, and lines indicate fitted values.

We tested for interactions among the associations of aging and MRFD with GMV. Introducing the product of age and the number of any of 4 MRFD (ie, hypertension, diabetes, obesity, and frequent alcohol use) for each individual improved the fit of the model (b = –0.39 mL; SE = 0.07 mL; *P* < .001), indicating that MRFD were associated with modifying the associations of age with total GMV. Figure 3 shows a steeper slope for the fit lines in the presence of a greater number of MRFD, suggesting that the association of age with change in total GMV was more pronounced when individuals were exposed to more MRFD (no MRFD, –0.45%/y; 1 MRFD, –0.51%/y; 2 MRFD, –0.56%/y; 3-4 MRFD, –0.60%/y).

### Associations of Cognitive Test Scores With Brain Regions Showing Volume Differences With Aging and MRFD

To better understand the potential clinical relevance of the GMV differences found in the generally healthy UKB population, we tested for voxelwise correlations in GMV with cognitive test performance (FWER-corrected *P* < .05). Better scores of spatial memory performance were positively correlated with greater GMV in the posterior cingulate cortex. Lower fluid intelligence test performance was associated with lower volume in the posterior cingulate cortex, the thalamus, and the orbitofrontal cortex, and reaction time was negatively associated with GMV in the thalamus and the hippocampus (eFigure 3 in the Supplement).

### Associations of Aging and MRFD With Cognitive Test Scores

We next tested for associations between aging and the 4 MRFD (ie, hypertension, diabetes, obesity, and frequent alcohol use) and differences in cognitive performance (eTable 2 in the Supplement). As expected, age was negatively associated with performance on tests of spatial memory (b = 0.06; SE = 0.004; *P* < .001), reaction time (b = 3.77; SE = 0.15; *P* < .001), and fluid intelligence (b = –0.03; SE = 0.003; *P* < .001). Participants reporting all 4 MRFD had lower spatial memory performance scores than those without any MRFD (b = 1.52; SE = 0.63; *P* = .048).

### Mediation Associated With Age or MRFD on Cognitive Performance by Brain Volume

**Figure 4.**
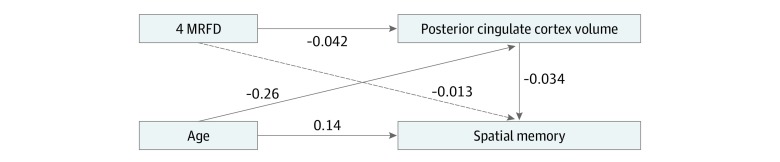
Structural Equation Model Exploring Associations Among Age, Modifiable Risk Factors for Dementia (MRFD), Posterior Cingulate Cortex Volume, and Spatial Memory Standardized correlation coefficients are shown. Solid lines indicate associations that were statistically significant; dashed line indicates no statistically significant association. The model had a χ^2^ of 0.000, root mean square error of approximation less than 0.001, Akaike information criterion of 103857.9, comparative fit index of 1.000, and Tucker-Lewis index of 1.000.

Based on the results of these brain volume and cognitive analyses, we used structural equation modeling to explore whether GMV in the posterior cingulate cortex could account for the associations of the 4 MRFD with lower spatial memory performance (Figure 4). There was no direct association of the 4 MRFD with spatial memory (β = –0.013; SE = 0.011; *P* = .23). However, the model showed that the negative associations of the 4 MRFD with spatial memory were associated with a partial mediation by lower GMV in the posterior cingulate cortex (β = 0.0014; SE = 0.0006; *P* = .02). There was a direct association of aging with spatial memory (β = 0.138; SE = 0.011; *P* < .001), and lower GMV mediated this association (β = 0.009; SE = 0.0004; *P* = .003).

## Discussion

This cross-sectional study explored brain structural differences associated with aging and MRFD in a middle-aged UKB population who did not report a history of neurological or psychiatric diseases. Independent associations of 4 MRFD (ie, hypertension, diabetes, obesity, and frequent alcohol use) with differences in GMV associated with age were discovered. Aging and the 4 MRFD were associated with distinct regional differences in GMV, suggesting differences in sensitivity. We hypothesized that these differences reflect sensitivity to the early pathology leading to AD. As hypothesized, we found that regions of lower GMV associated with aging and exposures to the 4 MRFD individually overlapped with each other and with regions of expected GMV loss associated with AD in the posterior cingulate cortex, the thalamus, the hippocampus, and the orbitofrontal cortex. The associations of aging and MRFD with GMV were synergistic, indicating that aging-related GMV loss may be more severe with more MRFD. The clinical relevance of even these small GMV differences was supported by the observation that volume differences in the posterior cingulate cortex were associated with mediation of the associations of age and the 4 MRFD with spatial memory performance.

Two previous population-based studies have mapped brain regions associated with risk factors for dementia. A 2004 study by Taki et al^13^ included 769 Japanese participants with no known cognitive disorders with information of systolic blood pressure, alcohol use, and smoking. In that cohort, age was negatively correlated with overall brain volume; associations of focal GMV with systolic blood pressure and alcohol use were found, but they did not overlap, possibly because of limitations of power with the smaller sample size.^13^

A 2019 study by Cox et al^20^ explored associations of multiple vascular risk factors with brain structure in a UKB population different from ours. Smoking pack-years, hypertension, and higher aggregate vascular risk were associated with lower volumes of several gray matter regions, including the frontal and temporal cortex, and in subcortical gray matter structures. However, despite exploring risk factors chosen for their association with vascular disease, a striking observation in that study was the relatively weak association with direct imaging evidence of cerebrovascular disease in the population (white matter hyperintense volumes on T2-weighted images or an anatomical distribution of gray matter volume differences in regions of brain characteristically associated with large or small vessel disease).^20,55,56^ The greatest cortical differences associated with the risk factors were found in the anterior temporal cortex. Differences in white matter diffusivity, a measure associated with axon or myelin pathologies, were found throughout the brain. In our study, we used voxelwise rather than atlas region–based analyses and more stringent statistical criteria^57^ to achieve greater neuroanatomical precision in mapping structural associations. We also investigated the more general hypothesis that risk factors for late-life cognitive impairment may be associated with AD independent of any associations with vascular disease. While our conclusions agree in suggesting regional associations with some risk factors, most of which are also found in the studies by Taki et al^13^ and Cox et al,^20^ we do not interpret this regional association as reflecting brain structural changes associated with hypoperfusion or stroke.

We made 3 novel observations. First, we observed associations of aging and 4 MRFD exposures with GMV differences in common regions of the posterior cingulate cortex, the thalamus, the hippocampus, and the orbitofrontal cortex that correspond well with regions of early AD neuropathology.^58,59,60,61^ Reduced metabolism in the posterior cingulate cortex may precede clinical expression of AD.^58^ The hippocampus and thalami both show early neurofibrillary changes and can develop pathology associated with core amnestic symptoms.^59,60^ Widespread neurofibrillary tangle pathology in the orbitofrontal cortex also is found in patients with AD.^61^

Second, associations of lower GMV with the MRFD were circumscribed within the larger brain regions showing a negative association of GMV with age. The additive contributions to AD risk from aging and these 4 MRFD^6,7^ may be explained by their anatomically overlapping independent associations with lower GMV. The GMV identified may have common associations with the apparently disparate risk factors through multiple mechanisms, such as involving local cell autonomous factor,^62^ properties of regional glial, dendritic, and synaptic networks or activity,^63,64^ or secondary associations mediated by longer range connectivities.^65^

Third, we provided indirect evidence for the relevance of these observations for late-life cognitive impairments in a relatively healthy middle-aged population. We showed the jointly overlapping associations of lower GMV with aging and the 4 MRFD were found within the larger volume of relative atrophy seen in an independent population with AD. Consistent with earlier studies, we found independent associations of age and the 4 MRFD with lower cognitive performance.^32,66,67,68,69^ We also found that variation in an overlapping posterior cingulate cortex GMV associated with aging and the 4 MRFD was associated with mediating the negative associations of these risk factors with spatial memory. This association of the relative volume of the posterior cingulate cortex with spatial memory is consistent with a previous report.^58^

### Limitations

This study had limitations, including that it is based on cross-sectional analyses. Effect sizes may be lower than some previous aging studies^10,15,16^ that included participants 80 years or older because our population was relatively young for the expression of late-life cognitive impairment. The UKB population also is generally healthier than the wider UK population.^35^ We limited our analyses to regional GMV because this is more directly interpretable than diffusion tensor-based measures of white matter connectivities or functional MRI measures, for example. There also is a larger literature in which to contextualize the results.^70,71^ Limitations of the data available meant that MRFD were treated categorically rather than by severity and years of exposure to risk; analyses based on the latter would be expected to enhance power to detect differences. Additionally, because of limitations of study power, we only tested for linear associations with aging or MRFD.

## Conclusions

This cross-sectional study found that aging and 4 MRFD, including hypertension, diabetes, obesity, and frequent alcohol use, were independently negatively associated with focal GMV in common regions of the posterior cingulate gyrus, the thalamus, the hippocampus, and the orbitofrontal cortex, which are also associated with the progression of AD. The associations observed in this generally healthy population highlight common regions of potential associations of risk factors with dementia and the association of these risk factors with mediation of their associations with reduced cognitive performance. We hypothesize that molecular pathology associated with the early biochemical phase of AD^72^ may be found in these regions. If the underlying mechanisms of association were identified and able to be targeted therapeutically, early stages of progression to AD could potentially be slowed or reversed.

## Notes

Supplement. **eAppendix.** Supplemental Methods **eReferences** **eFigure 1.** Distributions of Continuous Variables in the UK Biobank and ADNI Cohorts **eTable 1.** Correlation Coefficients in Multivariable Analysis for Age and Modifiable Risk Factors for Dementia **eFigure 2.** Gray Matter Regions With Volumes Associated With Sex **eFigure 3.** Associations of Cognitive Performance With Modifiable Factors-Related Brain Regions **eTable 2.** Associations of Cognitive Test Scores With Modifiable Risk Factors for Dementia and Age
