# Supplementary material for: Associations of Regional Brain Structural Differences With Aging, Modifiable Risk Factors for Dementia, and Cognitive Performance
Source: JAMA Netw Open. 2019 Dec 11;2(12):e1917257. doi: 10.1001/jamanetworkopen.2019.17257 (PMC6991214; doi:10.1001/jamanetworkopen.2019.17257)
Supplement: Supplement. — eAppendix. Supplemental Methods eReferences eFigure 1. Distributions of Continuous Variables in the UK Biobank and ADNI Cohorts eTable 1. Correlation Coefficients in Multivariable Analysis for Age and Modifiable Risk Factors for Dementia eFigure 2. Gray Matter Regions With Volumes Associated With Sex eFigure 3. Associations of Cognitive Performance With Modifiable Factors-Related Brain Regions eTable 2. Associations of Cognitive Test Scores With Modifiable Risk Factors for Dementia and Age [file jamanetwopen-2-e1917257-s001.pdf]

Supplementary Online Content

Suzuki H, Venkataraman AV, Bai W, et al; Alzheimer's Disease Neuroimaging Initiative. Associations of regional brain structural differences with aging, modifiable risk factors for dementia, and cognitive performance. *JAMA Netw Open*. 2019;2(12):e1917257. doi:10.1001/jamanetworkopen.2019.17257

eAppendix. Supplemental Methods
eReferences
eFigure 1. Distributions of Continuous Variables in the UK Biobank and ADNI Cohorts
eTable 1. Correlation Coefficients in Multivariable Analysis for Age and Modifiable Risk Factors for Dementia
eFigure 2. Gray Matter Regions With Volumes Associated With Sex
eFigure 3. Associations of Cognitive Performance With Modifiable Factors-Related Brain Regions
eTable 2. Associations of Cognitive Test Scores With Modifiable Risk Factors for Dementia and Age

This supplementary material has been provided by the authors to give readers additional information about their work.

© 2019 Suzuki H et al. *JAMA Network Open.*

eAppendix. Supplemental Methods

Modifiable risk factors for dementia (MRFD)

Details of the procedure for blood pressure measurement in UKB are available online.[1] We defined participants as hypertensive if they had systolic ≥140 or diastolic blood pressure ≥90mmHg or were receiving antihypertensive medication.[2] Participants were considered as obese if they had body mass index ≥30.[3,4] Diagnosis of diabetes, current smoking, frequency of alcohol drinking, sleep duration, and education attainment were all self-reported. Frequent alcohol consumption, inadequate sleep, and education attainment were defined as daily or almost daily alcohol drinking,[5] sleep duration of <6 or >8hrs,[6] and not achieving a college or university degree,[2] respectively. Numbers of the four MRFD showing overlap in associations in gray matter volume loss with Alzheimer's disease (hypertension, diabetes, obesity, and frequent alcohol consumption) also were used for analyses.

The dichortomized variables of MDRF can predict the risk of Alzheimer's disease. Hypertension (blood pressure greater than 140/90mmHg), obesity (body mass index greater than 30kg/m^2) and diabetes have higher relative risks of AD.[7,8] Although higher alcohol consumption is reported as a risk of AD,[9] the definitive abnormal ranges have not been decided like hypertension and obesity so we used daily or almost daily alcohol drinking in this study.

Brain MRI acquisition and pre-processing

Details of the image acquisition in UK Biobank (UKB) are available online.[10] MRI was acquired using a Siemens Skyra 3T running VD13A SP4 (Siemens Healthcare, Erlangen, Germany) with a Siemens 32-channel RF receive head coil. T1-weighted structural brain images were obtained using a three-dimensional MPRAGE sequence with a slice

© 2019 Suzuki H et al. *JAMA Network Open.*

thickness of 1mm and a field-of-view of 208×256×256mm.

MRI was acquired using either Siemens, GE, or Philips systems 3T scanners for images in the Alzheimer's Disease Neuroimaging Initiative (ADNI) resource. T1-weighted structural brain images were obtained using a three-dimensional MPRAGE sequence with a slice thickness of 1×1×1mm and a field-of-view of 208×240×256mm. Further technical details of image acquisition and standardization in ADNI have been described previously.[11,12]

Identical image processing was employed for analyses of the UKB and ADNI datasets using Statistical Parametric Mapping (SPM) 12 (Welcome, Department of Cognitive Neurology, London, UK) and custom-written software in Matlab (Math Works, Natick, MA, USA). To perform statistical analysis of the structural MRI images in the same standard space, spatial normalization was performed using voxel-based morphometry with diffeomorphic anatomical registration using exponentiated lie algebra (DARTEL),[13] as we previously described in detail.[14,15] Firstly, structural brain images were segmented to gray and white matter and cerebral spinal fluid probabilistic maps using a new segmentation algorithm. These tissue specific maps consisted of probability values ranging 0–1 (e.g. a voxel with gray matter probability value = 0.5 means it is 50% sure that this voxel is within gray matter). Intracranial volume (ICV), total brain volume, total gray matter volume (GMV), and total white matter volume were calculated from volume of the three tissue compartments.

Secondly, the 9932 segmented gray and white matter maps and those of 575 ADNI participants, including 100 Alzheimer’s disease (AD) patients, 127 late and 145 early mild cognitive impairment patients, 70 individuals with significant memory concern, and 133

© 2019 Suzuki H et al. *JAMA Network Open.*

cognitively normal people, were used to create a population-specific template for UKB and that for ADNI using the DARTEL template creation tool. The voxel size was sampled to 1.5×1.5×1.5mm to ensure less memory consumption. Third, the template space was matched to the standard Montreal Neurological Institute (MNI) space using an affine only registration. Finally, each participant's gray matter map was warped using its corresponding smooth and reversible deformation parameters to transform it to the custom template space and then to the standard MNI space.

The normalized gray matter maps were modulated with the Jacobian determinants and then were smoothed with an isotropic Gaussian kernel by convolving a 8-mm full width at half maximum to increase the validity of statistical inference in the voxel-based analysis. For voxel-wise analyses, we excluded voxels with a gray matter probability value below 0.2 to avoid possible edge effects between gray matter and white matter or cerebrospinal fluid.[14]

To illustrate the brain areas associated with AD, age, and each of the six MRFD to regional brain volume individually, surface meshes were made from brain volume associated with dementia, age, and MRFD and were rendered as a 3D mask on a transparent brain derived from the MRI template available in SPM 12, as described previously.[2]

Cognitive assessment

The methods for cognitive assessment were described previously.[16–19] For spatial memory test, participants were asked to memorize the positions of six card pairs, and then match them from memory while making as few errors as possible.[17] Scores on the pairs-matching test are for the number of errors that each participant made; therefore, higher

© 2019 Suzuki H et al. *JAMA Network Open.*

scores reflect poorer cognitive function. For reaction time test, participants completed a timed test of symbol matching, similar to the common card game 'Snap' hereafter referred to as reaction time.[18] The score on this task was the mean response time in milliseconds across trials which contained matching pairs. Fluid intelligence test was performed using thirteen logic/reasoning-type questions with a two-minute time limit.[19] The maximum score is 13.

Statistical analysis

Before the voxel-wise analysis, we explored the relative influences of age and MRFD on GMV globally. Total GMV was regressed onto age and six MRFD, including hypertension, diabetes, obesity, frequent alcohol consumption, current smoking, and inadequate sleep, adjusted for sex, ethnicity, educational attainment, and ICV at a significance threshold of $p<0.05$. Multi-collinearity was assessed by determining correlation coefficients between covariates: moderate correlations (0.65–0.66) between sex and ICV were found, while absolute values of those between the other covariates were less than 0.35 (**eTable 1**).

Before conducting structural equation modeling, we tested associations of cognitive test scores with MDRF-associated brain regions and those of aging and MRFD with cognitive test scores. We tested the first associations using a voxel-wise analysis: voxels within focal gray matter areas associated with dementia, age and the four MRFD (hypertension, diabetes, obesity, and frequent alcohol consumption) were regressed onto scores of spatial memory, reaction time, and fluid intelligence adjusted for age, number of the 4 MRFD, sex, ethnicity and ICV at the family-wise error rate-corrected significance threshold of $p<0.05$. To test the second association, associations of age or numbers of MRFD with cognitive test scores were tested by a regression model using scores of spatial memory,

© 2019 Suzuki H et al. *JAMA Network Open.*

reaction time and fluid intelligence as dependent variables and age and numbers of MRFD as independent variable adjusting for age, sex, and ethnicity at a significance threshold of $p<0.05$, corrected for multiple testing.

eReferences

1. UK Biobank. Blood Pressure. Version 1.0. UK Biobank. 2011. https://biobank.ctsu.ox.ac.uk/crystal/docs/Bloodpressure.pdf
2. Suzuki H, Gao H, Bai W, et al. Abnormal brain white matter microstructure is associated with both pre-hypertension and hypertension. *PLoS One.* 2017;12:e0187600.
3. Pannacciulli N, Del Parigi A, Chen K, Le DS, Reiman EM, Tataranni PA. Brain abnormalities in human obesity: a voxel-based morphometric study. *Neuroimage.* 2006;31:1419–1425.
4. Taki Y, Kinomura S, Sato K, et al. Relationship between body mass index and gray matter volume in 1,428 healthy individuals. *Obesity (Silver Spring).* 2008;16:119–124.
5. Smith DJ, Nicholl BI, Cullen B, et al. Prevalence and Characteristics of Probable Major Depression and Bipolar Disorder within UK Biobank: Cross-Sectional Study of 172,751 Participants. *PLoS One.* 2013;8:e75362.
6. Tsai TC, Wu JS, Yang YC, Huang YH, Lu FH, Chang CJ. Long sleep duration associated with a higher risk of increased arterial stiffness in males. *Sleep.* 2014;37:1315–1320.
7. Norton S, Matthews FE, Barnes DE, Yaffe K, Brayne C. Potential for primary prevention of Alzheimer's disease: an analysis of population-based data. *Lancet Neurol.* 2014;13:788–794.
8. Walker KA, Sharrett AR, Wu A, et al. Association of Midlife to Late-Life Blood

© 2019 Suzuki H et al. *JAMA Network Open.*

Pressure Patterns With Incident Dementia. *JAMA.* 2019;322:535–545.

9. Harwood DG, Kalechstein A, Barker WW, et al. The effect of alcohol and tobacco consumption, and apolipoprotein E genotype, on the age of onset in Alzheimer's disease. *Int J Geriatr Psychiatry.* 2010;25:511–518.
10. UK Biobank. Brain Imaging Documentation. Version 1.2. UK Biobank. 2016. https://biobank.ctsu.ox.ac.uk/crystal/docs/brain_mri.pdf
11. Wyman BT, Harvey DJ, Crawford K, et al. Standardization of analysis sets for reporting results from ADNI MRI data. *Alzheimers Dement.* 2013;9:332–337.
12. Jack CR Jr, Barnes J, Bernstein MA, et al. Magnetic resonance imaging in Alzheimer's Disease Neuroimaging Initiative 2. *Alzheimers Dement.* 2015;11:740–756.
13. Ashburner J. A fast diffeomorphic image registration algorithm. *Neuroimage.* 2007;38:95–113.
14. Suzuki H, Sumiyoshi A, Matsumoto Y, et al. Structural abnormality of the hippocampus and depressive symptoms in heart failure rats. *NeuroImage.* 2015;105:84–92.
15. Suzuki H, Matsumoto Y, Ota H, et al. Hippocampal Blood Flow Abnormality Associated With Depressive Symptoms and Cognitive Impairment in Patients With Chronic Heart Failure. *Circ J.* 2016;80:1773–1780.
16. Lyall DM, Cullen B, Allerhand M, et al. Cognitive Test Scores in UK Biobank: Data Reduction in 480,416 Participants and Longitudinal Stability in 20,346 Participants. *PLoS One.* 2016;11:e0154222.
17. UK Biobank. Touch-screen Pairs memory test. Version 1.2. UK Biobank. 2013. biobank.ctsu.ox.ac.uk/crystal/docs/Pairs.pdf
18. UK Biobank. Touch-screen test of reaction time (Snap). Version 1.1. UK Biobank. 2015. biobank.ctsu.ox.ac.uk/crystal/docs/Snap.pdf

© 2019 Suzuki H et al. *JAMA Network Open.*

19. UK Biobank. Touch-screen Fluid intelligence test. Version 1.1. UK Biobank. 2012. biobank.ctsu.ox.ac.uk/crystal/docs/Fluidintelligence.pdf

© 2019 Suzuki H et al. *JAMA Network Open.*

eFigure 1. Distributions of Continuous Variables in the UK Biobank and ADNI Cohorts

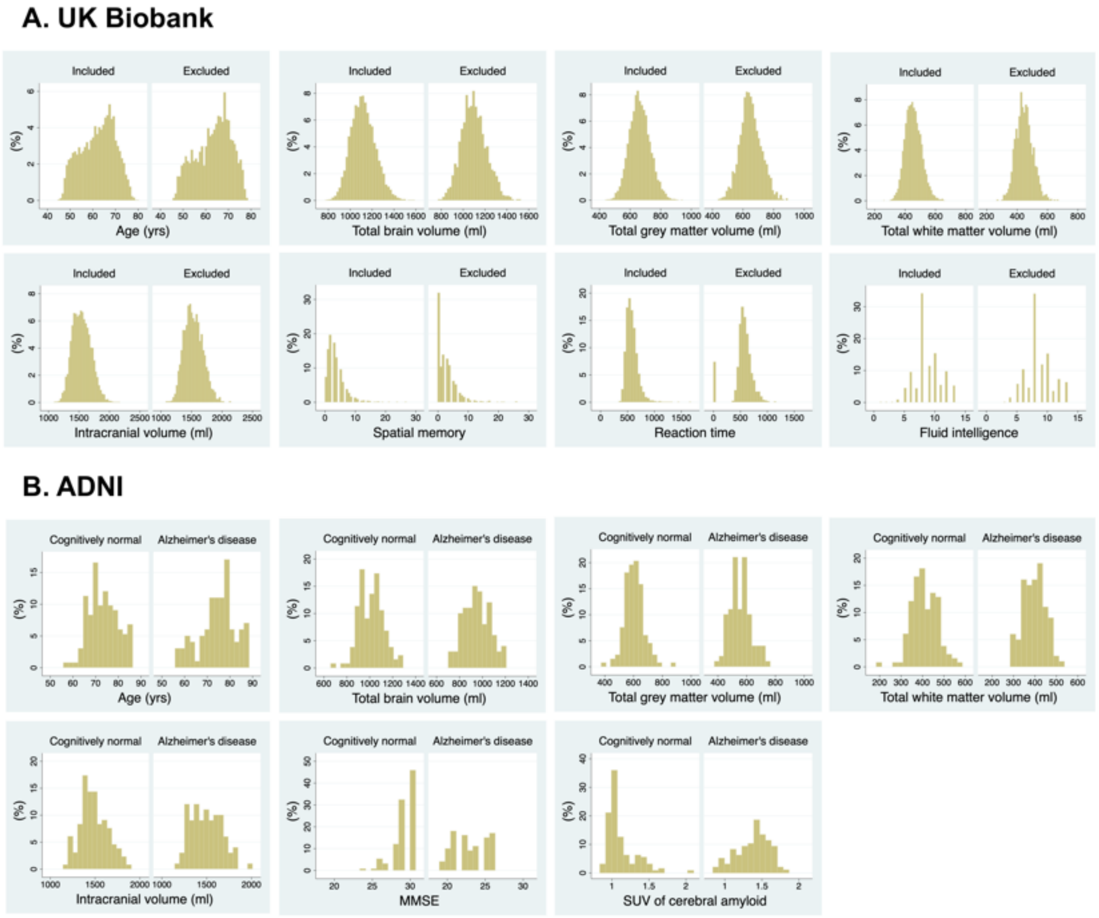

© 2019 Suzuki H et al. *JAMA Network Open.*

eTable 1. Correlation Coefficients in Multivariable Analysis for Age and Modifiable Risk Factors for Dementia

	age	sex	ethnicity	intracranial volume	hypertension	diabetes	obesity	smoking	alcohol	sleep	education
age	1.00										
sex	0.09	1.00									
ethnicity	0.09	0.00	1.00								
intracranial volume	0.04	0.66	0.08	1.00							
hypertension	0.27	0.19	0.03	0.10	1.00						
diabetes	0.08	0.07	–0.03	0.03	0.13	1.00					
obesity	–0.04	0.01	0.00	0.01	0.17	0.15	1.00				
smoking	0.10	0.10	0.05	0.09	0.06	–0.02	–0.04	1.00			
alcohol	–0.09	0.04	–0.02	0.02	–0.03	0.00	–0.01	0.05	1.00		
sleep	0.04	–0.02	0.00	–0.03	0.02	0.03	0.05	0.02	0.02	1.00	
education	0.07	–0.05	0.04	–0.11	0.06	0.04	0.07	–0.06	0.01	0.06	1.00

© 2019 Suzuki H et al. *JAMA Network Open.*

eFigure 2. Gray Matter Regions With Volumes Associated With Sex

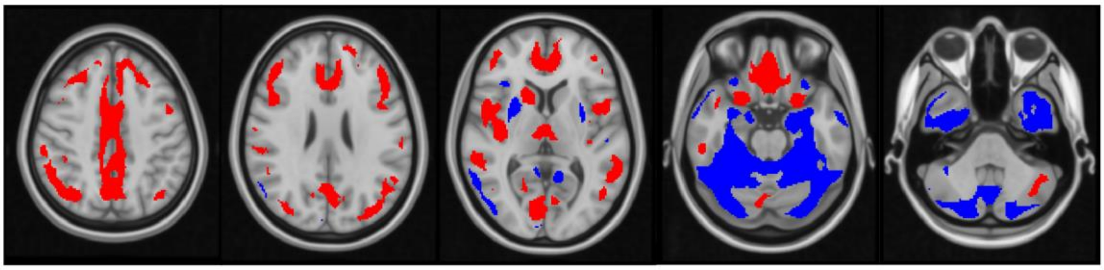

Gray matter regions showing lower (red) and higher (blue) volume in men relative to women in UK Biobank. The results were derived from the same voxel-wise regression model for age and modifiable risk factors for dementia. The results exceeded a significance threshold of $p<.05$ with family-wise error rate corrections and were displayed on the same slices as **Figure 1** and **2** (43.5mm, 25.5mm, 6.0mm, –22.5mm and –40.5mm from the bregma) from the MNI152_T1_0.5mm template available FSLeyes.

© 2019 Suzuki H et al. *JAMA Network Open.*

eFigure 3. Associations of Cognitive Performance With Modifiable Factors-Related Brain Regions

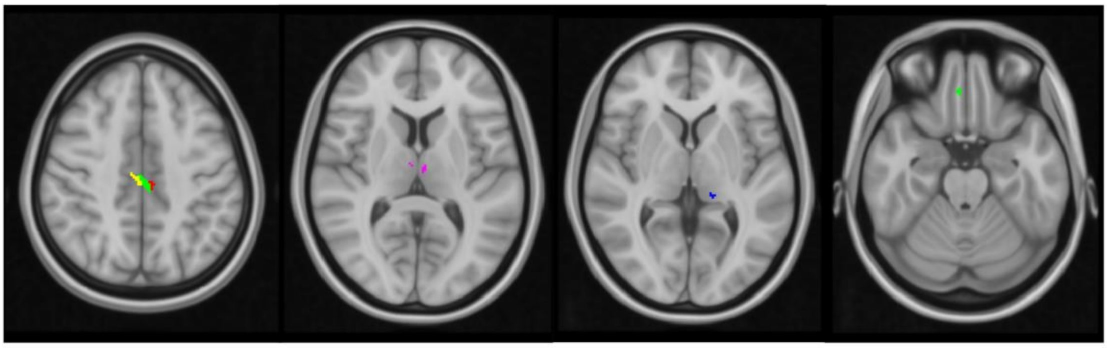

Gray matter regions whose volume was associated with spatial memory (red), fluid intelligence (green), reaction time (blue) and both reaction time and fluid intelligence (mazenda). Yellow areas indicate volumes associated with hypertension, diabetes, obesity and frequent alcohol consumption but not with any cognitive test scores. The results exceeded a significance threshold of $p<.05$ with family-wise error rate corrections and were displayed on the axial slices of 43.5mm, 25.5mm, 6.0mm and –22.5mm from the bregma from the MNI152_T1_0.5mm template available FSLeyes.

© 2019 Suzuki H et al. *JAMA Network Open.*

eTable 2. Associations of Cognitive Test Scores With Modifiable Risk Factors for Dementia and Age

	Spatial memory score			Reaction time (ms)			Fluid intelligence score		
	Estimate	Standard error	*p* value	Estimate	Standard error	*p* value	Estimate	Standard error	*p* value
*Age (yrs)	0.06	0.004	<.001	3.77	0.15	<.001	–0.03	0.003	<.001
†Numbers of 4 modifiable factors									
0 (N=3073)	Reference			Reference			Reference		
1 (N=3343)	–0.06	0.07	1.00	–1.31	2.55	1.00	0.09	0.05	0.231
2 (N=1572)	–0.13	0.09	0.38	0.52	3.19	1.00	0.05	0.07	1.000
3 (N=305)	–0.19	0.17	0.69	4.67	6.03	1.00	0.06	0.12	1.000
4 (N=19)	1.52	0.63	0.048	30.7	23.9	0.54	–0.73	0.47	0.363

*Estimates for age indicate differences in cognitive test scores if age increases by 1 year. †Estimates for numbers of modifiable factors indicate differences relative to the references.

© 2019 Suzuki H et al. *JAMA Network Open.*
